# Current state-of-the-art for allergen immunoassays

**DOI:** 10.1186/2045-7022-1-S1-S80

**Published:** 2011-08-12

**Authors:** Franz Ulberth

**Affiliations:** 1 European Commission - Joint Research Centre, Institute for Reference Materials and Measurements, Geel, Belgium

## 

A considerable proportion of the general public experiences negative health effects triggered by certain allergens contained in their habitual diet. Usually the recommended option is to eliminate from the diet the food ingredients to which hypersensitivity has been found. Allergic persons need to know whether the food items they purchase contain them and they have to rely on the truthfulness of information given on the label of packaged food items. Legislation has been put in place which requires food business operators to declare whether ingredients with a known allergenic potential have been used during manufacturing. Analytical testing systems are needed by the food industry to enable them to test whether allergens are present in their raw materials, the finished products and whether production lines have been correctly sanitised, by the food inspection authorities for market surveillance and by academia to enable and stimulate research into food allergy and allergen detection. Immunochemistry based tests, and in particular ELISAs, are definitely the most widely applied tool in routine as well as research laboratories for the determination of food allergens. Lateral flow devices are, in terms of simplicity of application and user friendliness, probably the most advanced analytical tools for the rapid detection of allergens. They are widely applied in the food manufacturing industries for the control of production lines. Food processing can have a profound impact on the detectability of allergens by ELISA based methods. Advantages but also the inherent disadvantages of ELISA tests, and in particular the importance of validating such methods properly by including naturally incurred test materials in validation exercises, are important aspects, which have to be addressed in an appropriate manner by test developers, end-users and validation/standardisation bodies. Comparability of testing results produced by different assays is not always ensured, and approaches how to achieve this (e.g. clearer definition of what entity is measured, use of well characterised materials for calibration, etc) should be explored to improve the equivalence of testing results.

